# EXPLORING CONGRUENCY OF DEATH LOCATION PREFERENCES VERSUS EXPECTATIONS: FINDINGS FROM BRAZIL, ITALY, JAPAN, AND THE US

**DOI:** 10.1093/geroni/igad104.1888

**Published:** 2023-12-21

**Authors:** Sarah Clem, Peiyuan Zhang, John Cagle

**Affiliations:** University of Maryland School of Social Work, Baltimore, Maryland, United States; University of Maryland, Baltimore, Baltimore, Maryland, United States; University of Maryland, Baltimore, Baltimore, Maryland, United States

## Abstract

Dying in one’s preferred death location is a common indicator of a good death. Previous studies exploring the congruency between individuals’ death location wishes and their actual death locations relied on samples of patients with terminal diagnoses. Data for this study came from the Four-Country Survey on Aging and End-of-Life Medical Care 2017 from which nationally representative samples of the general public were collected in the U.S., Italy, Japan, and Brazil. Bivariate analyses of the total sample (n=4,239) identified that 45% of participants reported expected death locations that differed from their preferred place of death. Among the majority of participants reporting a preference for a home death, 61% of these individuals in Japan (p<.001) and 43% from Brazil (p<.001) reported they were more likely to die in a hospital. Whereas, in both Italy and the U.S., the majority of participants within each death location category reported the same location for their preferred and most likely death location. Logistic regression models identified unique predictors of congruency for each country. Noteworthy, in the Italy model, females were 43% lower odds of having congruent death location perceptions compared to men (OR=0.57, p=0.02). In the U.S. model, racial differences were also notable, as Black participants had over 2 times greater odds of congruent death location perceptions compared to White participants (OR=2.18, p=0.02). By further understanding the congruency of death location perceptions among the general public, we can gain insight into the perception of whether individuals feel that a quality death experience is attainable.

